# Supplementary material for: The importance of relativistic effects on two-photon absorption spectra in metal halide perovskites
Source: Nat Commun. 2019 Nov 25;10:5342. doi: 10.1038/s41467-019-13136-y (PMC6877591; doi:10.1038/s41467-019-13136-y)
Supplement: Supplementary file 1 — Supplementary Information [file 41467_2019_13136_MOESM1_ESM.pdf]

# Supplementary information for

# The Importance of Relativistic Effects on Two-Photon Absorption Spectra in Metal Halide Perovskites

Wei et al.

Supplementary Figure 1-10

Supplementary Table 1

Supplementary Note 1-5

Supplementary References

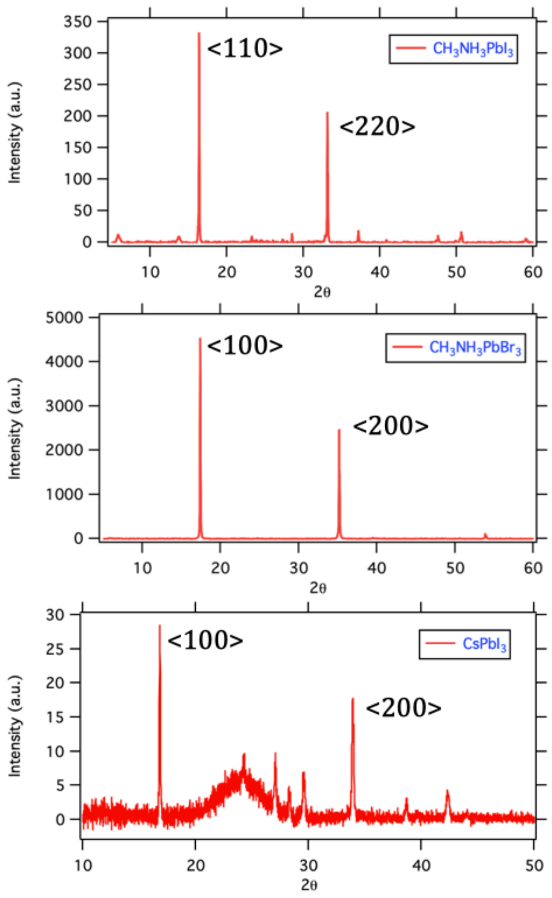


**Supplementary Figure 1**. XRD pattern for (a) $CH_3NH_3PbI_3$ (b) $CH_3NH_3PbBr_3$ and (c) $CsPbI_3$ thin film (Co *κ*α radiation, λ = 1.78 Å).

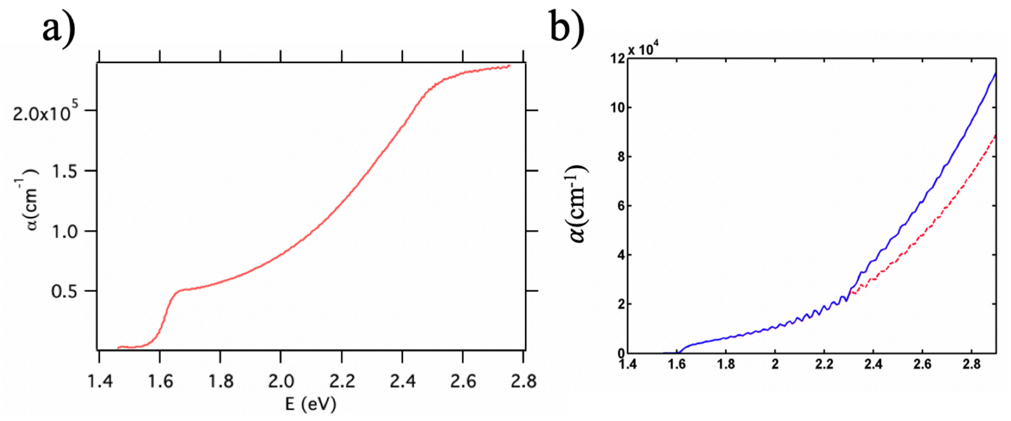


**Supplementary Figure 2**. (a) Experimental 1PA spectrum of $CH_3NH_3PbI_3$ thin film. (b) 1PA spectrum computed using the empirical tight binding model. The red dashed line represents the 1PA spectrum computed by considering only the optical transitions to the bottom of the conduction band (spin-orbit split-off bands). The difference between the two spectra evidences the contribution of the heavy and light electron bands.

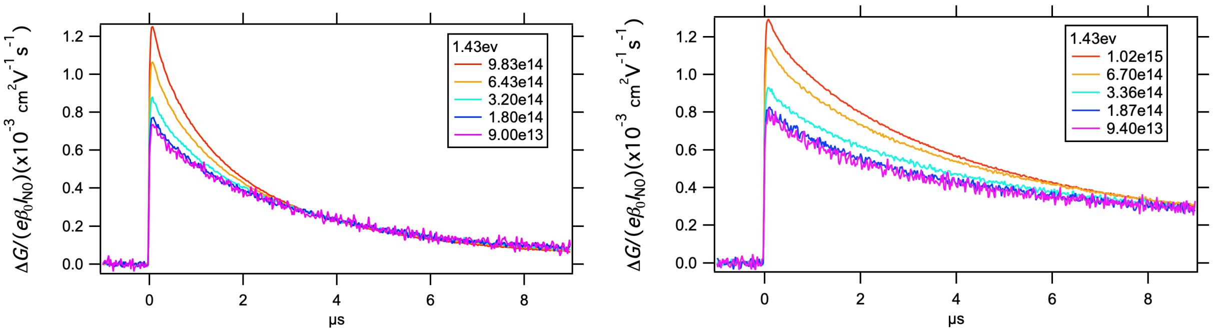


**Supplementary Figure 3**. TRMC traces for a $CH_3NH_3PbI_3$ thin film measured before (left) and after (right) the light soaking treatment at excitation photo energy of 1.43 eV (867nm). The legend in each graph shows incident light intensity in photons $cm^{-2}$.

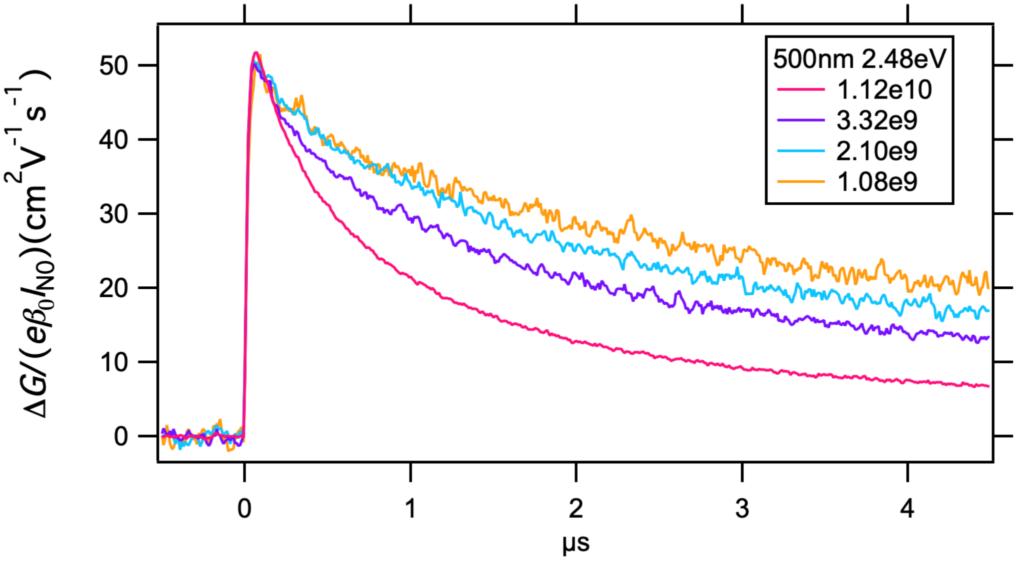


**Supplementary Figure 4**. Intensity normalized photoconductance as a function of time above the bandgap energy (500 nm) under different intensities. The legend shows incident light intensity in photons $cm^{-2}$.

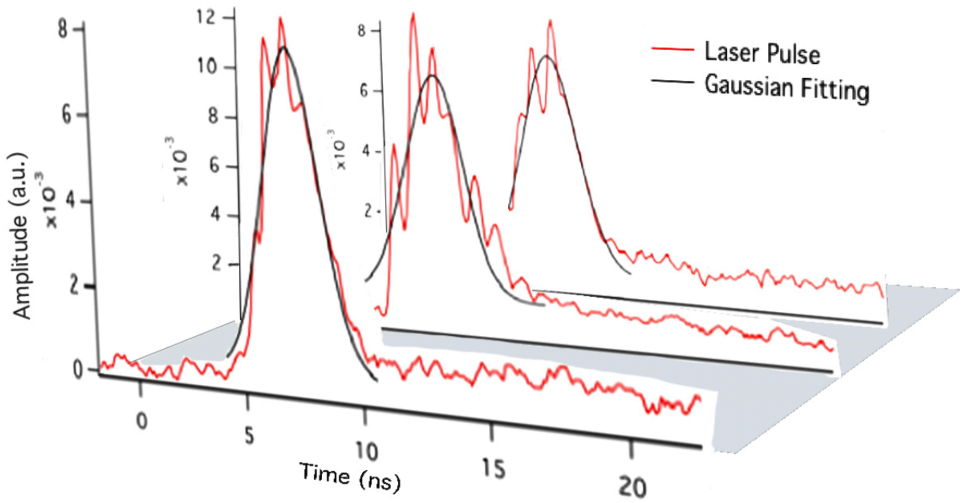


**Supplementary Figure 5**. Laser pulse traces recorded at 900 nm and corresponding Gaussian fitting.

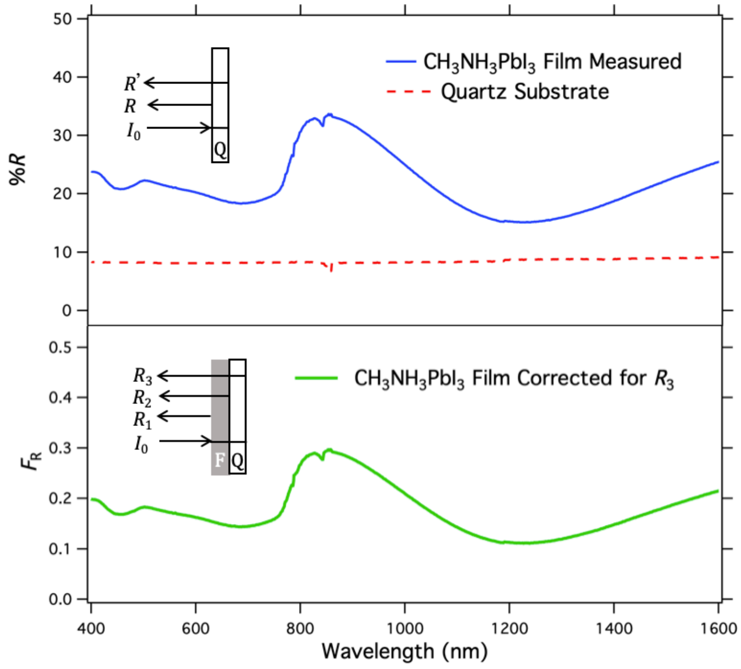


**Supplementary Figure 6**. The reflectance for a $CH_3NH_3PbI_3$ thin film deposited on the substrate (blue curve) and a quartz substrate (dashed line) and the fraction of reflected light for the thin film (green curve).

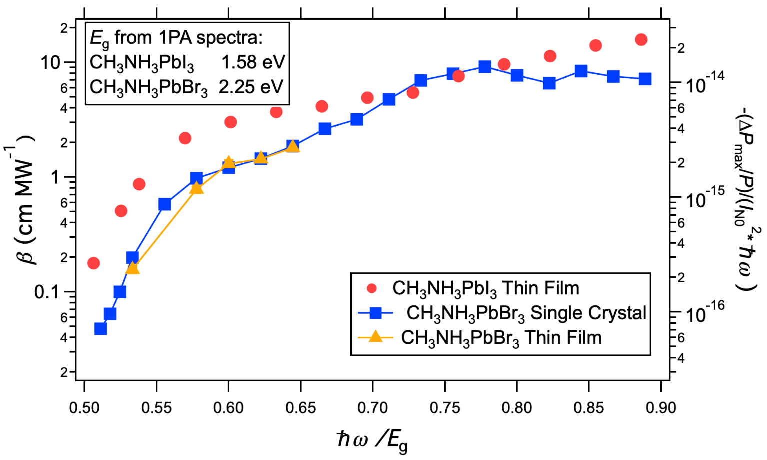


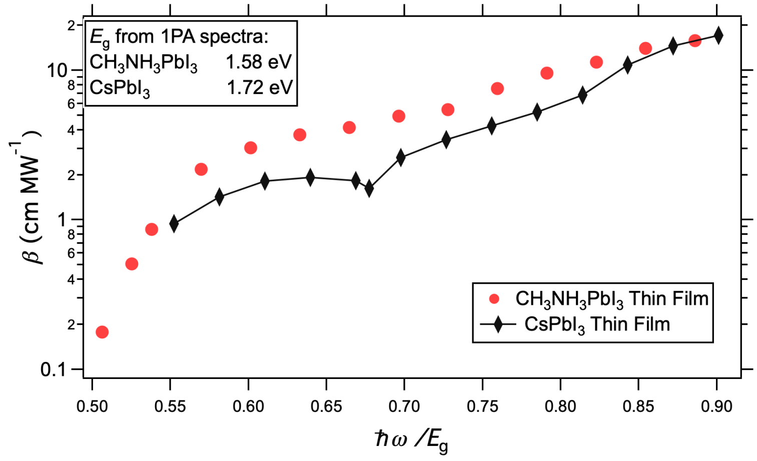


**Supplementary Figure 7**. 2PA spectra for $CH_3NH_3PbBr_3$ thin film and single crystal (top) and $CsPbI_3$ thin film (bottom). 2PA spectrum for $CH_3NH_3PbI_3$ is used as a reference.

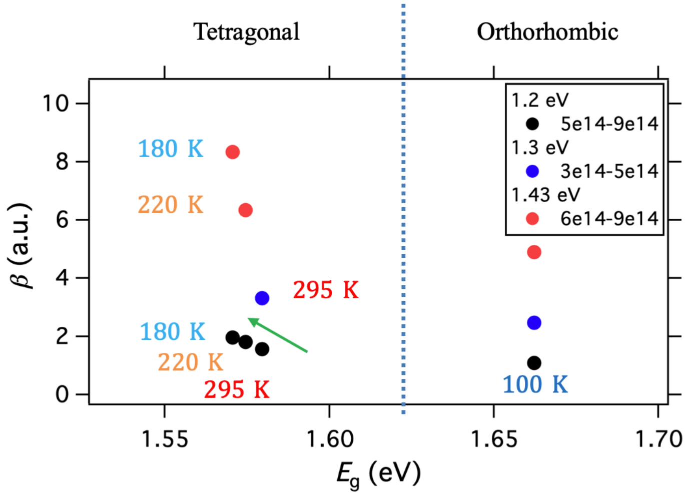


**Supplementary Figure 8.** 2PA coefficient $\beta$ of $CH_3NH_3PbI_3$ *versus* the bandgap energy $E_g$ recorded at different excitation energies (1.2 eV, 1.3 eV, and 1.43 eV). Legend shows the incident light intensity $I_{N0}$ in photons $cm^{-2}$ per pulse. Corresponding data are provided in **Supplementary Table 1**.

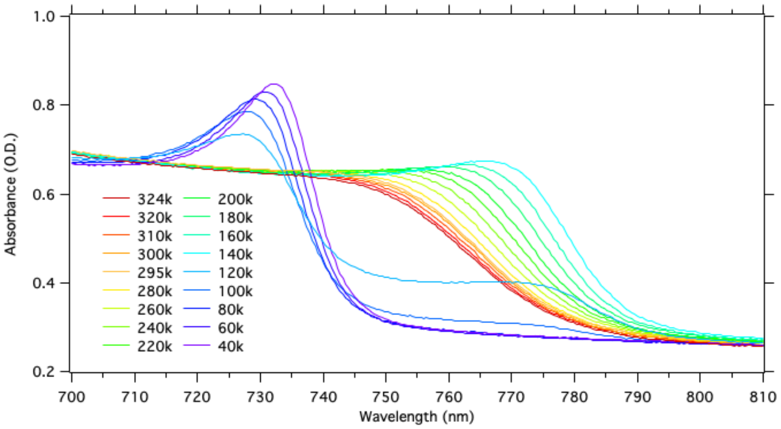


**Supplementary Figure 9**. Temperature-dependent absorbance for $CH_3NH_3PbI_3$ thin film.

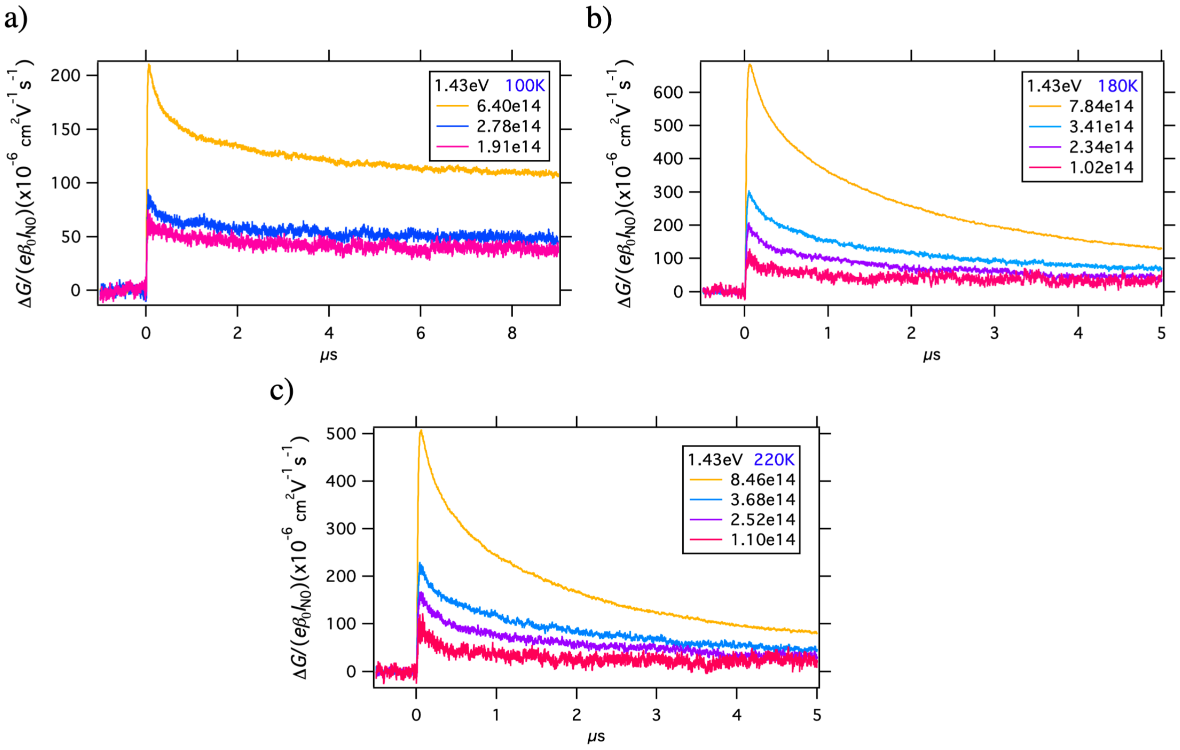


**Supplementary Figure 10.** TRMC traces for a $CH_3NH_3PbI_3$ thin film at excitation energy of 1.43 eV measured at (a) 220K, (b) 180K and (c) 100K. The legend in each graph shows incident light intensity $I_{N0}$ in photons cm$^{-2}$.

**Supplementary Table 1**. **Two photon absorption coefficients**

2PA coefficient $\beta$ of $CH_3NH_3PbI_3$ using temperature-dependent TRMC recorded for $\hbar\omega$ = 1.2 eV, 1.3 eV, and 1.43 eV, which were determined at $I_{N0}$ varying from $5 \times 10^{14}$ to $9 \times 10^{14}$ photons cm$^{-2}$ per pulse. The value of $E_g$ at each temperature was determined by the cut-off wavelength from the temperature-dependent linear absorption spectra (**Supplementary Figure 9**). The values of $\beta$ calculated from the temperature-dependent TRMC measurements are set as arbitrary unit due to the near infrared sensitivity of the temperature-dependent TRMC cell.

| $T$ (K) | $E_g$ (eV) | $\hbar\omega$ (eV) | | |
|---|---|---|---|---|
| | | 1.2 | 1.3 | 1.43 |
| 180 | 1.5706 | **1.96** | **N/A** | **8.34** |
| 220 | 1.5746 | **1.81** | **N/A** | **6.35** |
| 295 | 1.5796 | **1.57** | **3.31** | **N/A** |
| 100 | 1.6622 | **1.09** | **2.47** | **4.89** |

**Supplementary Note 1.**

In order to analyze the 2PA spectrum, we used the empirical tight binding model of **Supplementary** Reference 1, slightly modified to obtain a better match with the experimental 2PA spectrum. The rate of photon absorption per volume, V, associated to a band to band 1PA (neglecting excitonic interaction) between an initial valence band state v and a final conduction band state c at the $\mathbf{k_v}, \mathbf{k_c}$ points in the Brillouin zone may be computed from the first order Fermi golden rule:

$$\mathrm{R}_{cv} = \frac{2}{V}\sum_{\boldsymbol{k}_v}\sum_{\boldsymbol{k}_c}\frac{2\pi}{\hbar}|H^{cv}|^2\delta(E_c - E_v - \hbar\omega) \qquad (1)$$

where the matrix element $H^{cv} = \frac{-eA_0}{2m_0}\boldsymbol{e}.\boldsymbol{p}^{cv}$ and $\boldsymbol{p}^{cv} = \langle c|\hat{p}|v\rangle$. The absorption coefficient is given by:

$$\alpha^L_{cv} = \frac{R_{cv}}{S/\hbar\omega} = \frac{2\hbar\omega}{n_r c\varepsilon_0\omega^2 A_0^2}R_{cv} \qquad (2)$$

where S is the time average of the Poynting vector. Next, neglecting excitonic interaction between the conduction and valence band states, as well as the photon wavevector ($\mathbf{k}_{\mathrm{ph}} \approx 0, \mathbf{k_v} = \mathbf{k_c} = \mathbf{k}$), the absorption coefficient reads:

$$\alpha^L \approx \frac{C_L}{V}\sum_{c}\sum_{v}\sum_{\boldsymbol{k}}|\boldsymbol{e}.\boldsymbol{p}^{cv}|^2\delta(E_c - E_v - \hbar\omega) \qquad (3)$$

where $C_L = \frac{\pi e^2}{n_r c\varepsilon_0\omega m_0^2}$ and $\boldsymbol{p}^{cv} = \int u^*_{c,k}\widehat{\boldsymbol{p}}u_{v,\boldsymbol{k}}d^3\boldsymbol{r}$ is the Kane parameter computed for the two corresponding Bloch functions. Such an expression allows to performing the integration over the entire Brillouin zone within the tight-binding approach. Introducing $\widehat{\boldsymbol{p}} = \frac{-im_0}{\hbar}[\widehat{\boldsymbol{H}}, \widehat{\boldsymbol{r}}]$, the

momentum matrix elements $\boldsymbol{p}^{cv}$ can be computed from summation over the matrix elements $\boldsymbol{p}(\boldsymbol{k}) = \frac{m_0}{\hbar}\overrightarrow{\boldsymbol{\nabla}_{\boldsymbol{k}}}\boldsymbol{H}(\boldsymbol{k})$, where $\boldsymbol{H}(\boldsymbol{k})$is the tight-binding Hamiltonian developed over a basis of atomic orbitals including spin-orbit coupling.

Interestingly, the computed 1PA spectrum exhibits an additional contribution above 2.3 eV attributed to the heavy (HE) and light electron (LE) bands (**Supplementary Figure 2b**). In a similar way, the band to band 2PA can be computed from the second order Fermi golden rule:

$$\alpha^{NL} \approx \frac{C_{NL}}{V}\sum_{c}\sum_{v}\sum_{\boldsymbol{k}}\left|\sum_{s}\frac{\boldsymbol{e}.\boldsymbol{p}^{cs}\boldsymbol{e}.\boldsymbol{p}^{sv}}{E_s - E_v - \hbar\omega}\right|^2 \delta(E_c - E_v - 2\hbar\omega) \qquad (4)$$

where s is an intermediate virtual state and $C_{NL} = \frac{\pi e^4 A_0^2}{4n_r c\varepsilon_0\omega m_0^4}$ .

Finally, **Supplementary Equation 5** yields the 2PA coefficient, $\beta$

$$\beta = \frac{2\alpha^{NL}}{S} \qquad (5)$$

**Supplementary Note 2.**

It has been reported that the fast decay process, such as second-order recombination, could reduce the lifetime of charge carriers ($\tau_{1/2}$) as well as the maximum intensity-normalized TRMC signal due to the instrumental response time of 18 ns[2]. By treating the sample with the light-soaking in air, $\tau_{1/2}$ in the sample was prolonged by a factor of 2 for the absorption below the bandgap (**Supplementary Figure 3**). Nevertheless, no significant changes in the maximum signals were observed after the light soaking treatment. It is thereby reasonable to assume that the recombination process is negligible at the initial measuring time.

**Supplementary Note 3.**

A silicon photodetector was used to record the pulse signals as a function of time. For each wavelength, ten shots were measured and fitted by a Gaussian function. The measurements can hold for different intensities, because the filters used to vary the laser intensity will not change the pulse shape. **Supplementary Figure 5** shows three examples of laser pulse traces measured at 900 nm and the corresponding Gaussian fitting. Results show that the average pulse duration is around $3.0 \pm 0.2$ ns for the wavelengths of interest. Although only wavelengths less than 1100 nm can be well characterized due to the limitation of the photodetector, it is reasonable to assume that a laser pulse of a longer wavelength has the similar pulse profile as its co-generated counterpart in the visible regime.

**Supplementary Note 4.**

The reflectance of the sample and the quartz substrate in the NIR regime was measured by the UV/Vis/NIR spectrometer (**Supplementary Figure 6**). As light travels through a quartz substrate in the air, reflections will occur at the two interfaces of the refractive index change. For the case of normal incidence (See in the upper inset), both surfaces of the non-absorbing quartz substrate should contribute equally to the reflectance according to the Fresnel's equation:

$$R = \frac{(n_1 - n_2)^2}{(n_1 + n_2)^2} \qquad (6)$$

Where the reflection is determined by the refractive index of the first and the second medium ($n_1$, $n_2$). For a thin film deposited on a quartz substrate (See in the lower inset), the total reflection is approximately to be comprised of three parts, the reflection at the interface between air and thin film, $R_1$, thin film and the substrate, $R_2$, and the substrate and air, $R_3$. The refractive index of air and quartz are known to be around 1.0 and 1.5, respectively[3,4]. Typically, a $CH_3NH_3PbI_3$ thin film has a refractive index of ~ 2.5 in the NIR regime[5]. Hence, the reflection mainly stems from the interface between air and film, while $R_2$ and $R_3$ have comparable smaller contributions to the total reflection. **Supplementary Figure 6** shows that the total reflectance from both surfaces of the substrate is almost a constant of 8%, while the reflectance of the sample varies with the excitation wavelengths. Since most of light is not absorbed by the film in this regime, $R_3$ is estimated to be 4%. $R_1$ and $R_2$ are associated with the wavelength-dependent refractive index of the $CH_3NH_3PbI_3$ film, which is difficult to be measured separately. Therefore, the fraction of reflected light, $F_R$ in the sample was only corrected for the rear surface of the quartz substrate. In conjunction with the

laser power measured by the power meter, the incident light intensity $I_{\mathrm{N0}}$ entering the sample can be obtained.

**Supplementary Note 5.**

As shown in **Supplementary Figure 7**, both $CH_3NH_3PbBr_3$ thin film and single crystal exhibit the 2PA behavior at photon energies near 0.5 $E_g$. No clear quadratic dependence on intensity was observed for the $CH_3NH_3PbBr_3$ thin film in the higher energy regime ($\hbar\omega > 1.5$ eV). Instead, the lowest intensity leads to the highest photogenerated signal suggesting the saturable absorption of the sub-bandgap levels. The $CH_3NH_3PbBr_3$ single crystal demonstrated clear 2PA behavior over a wide range of energies, as not until 2.1 eV was the SLA detected. For the $CsPbI_3$ thin film, the second contribution was observed at 0.7 $E_g$. All spectra have taken into account the reflection and the sample thickness. Note that the $CsPbI_3$ sample was prepared by vapor deposition.

**Supplementary References:**


1. Boyer-Richard, S. *et al.* Symmetry-Based Tight Binding Modeling of Halide Perovskite Semiconductors. *J. Phys. Chem. Lett.* **7**, 3833-3840 (2016).

2. Hutter, E. M., Eperon, G. E., Stranks, S. D. & Savenije, T. J. Charge carriers in planar and meso-structured organic–inorganic perovskites: mobilities, lifetimes, and concentrations of trap states. *J. Phys. Chem. Lett.* **6**, 3082–3090 (2015).

3. Ciddor, P. E. Refractive index of air: new equations for the visible and near infrared. *Appl. Opt.* **35**, 1566-1573. (2008).

4. Rodríguez-de Marcos, L. V., Larruquert, J. I., Méndez, J. A. & Aznárez, J. A. Self-consistent optical constants of $SiO_2$ and $Ta_2O_5$ films. *Opt. Mater. Express* **6**, 3622-3637. (2016).

5. Phillips, L. J. *et al.* Dispersion relation data for methylammonium lead triiodide perovskite deposited on a (100) silicon wafer using a two-step vapour-phase reaction process. *Data Br.* **5**, 926-928(2015).
